# Zebrafish as an animal model for biomedical research

**DOI:** 10.1038/s12276-021-00571-5

**Published:** 2021-03-01

**Authors:** Tae-Young Choi, Tae-Ik Choi, Yu-Ri Lee, Seong-Kyu Choe, Cheol-Hee Kim

**Affiliations:** 1 Department of Pathology, Digestive Disease Research Institute, Wonkwang University, Iksan, Jeonbuk 54538 Republic of Korea; 2 Department of Biomedical Science, Graduate School, Wonkwang University, Iksan, Jeonbuk 54538 Republic of Korea; 3 Department of Biology, Chungnam National University, Daejeon, 34134 Republic of Korea; 4 Department of Microbiology, Wonkwang University, Iksan, Jeonbuk 54538 Republic of Korea; 5 Institute of Wonkwang Medical Science, Wonkwang University, Iksan, Jeonbuk 54538 Republic of Korea

**Keywords:** Disease model, Zebrafish

## Abstract

Zebrafish have several advantages compared to other vertebrate models used in modeling human diseases, particularly for large-scale genetic mutant and therapeutic compound screenings, and other biomedical research applications. With the impactful developments of CRISPR and next-generation sequencing technology, disease modeling in zebrafish is accelerating the understanding of the molecular mechanisms of human genetic diseases. These efforts are fundamental for the future of precision medicine because they provide new diagnostic and therapeutic solutions. This review focuses on zebrafish disease models for biomedical research, mainly in developmental disorders, mental disorders, and metabolic diseases.

## Animal models: Zebrafish help unlock clues to human disease

With their see-through bodies, low maintenance costs and genetic similarity to humans, zebrafish provide a powerful animal model for studying mental disorders and metabolic diseases in the laboratory. Tae-Young Choi from Wonkwang University, Iksan, South Korea, and coworkers review the many physiological advantages and logistical benefits of rearing these small tropical fish for biomedical research. These include the ease of tissue imaging, the large number of offspring in each generation and the increasing number of genetic techniques available. The researchers highlight the various ways in which zebrafish have contributed to scientists’ understanding of mental disorders and the communication pathways between brain and other organs in the body. They also discuss the potential of zebrafish for tracking metabolism and how it can go awry in various disease settings.

## Introduction

For use in genetic studies as an animal model, zebrafish was initially introduced by Streisinger and colleagues^1^ in the early 1980s. Large-scale N-ethyl-N-nitrosourea (ENU) mutagenesis was conducted in combination with extensive phenotypic screening^2,3^. Further phenotypic characterization of these ENU mutations in most of the major organ systems was performed^4^. However, later positional cloning of each ENU mutation after a forward genetic screen was time-consuming and laborious^5^. Since the increase in resolution of the zebrafish genome map, advanced gene-targeting technologies involving ZFNs, TALENs, and CRISPR/Cas9^6–12^, have overcome challenges in generating specific gene-knockout mutations. CRISPR/Cas9 utilizes an efficient reverse genetic approach to provide knockout animals for zebrafish researchers^13^. Furthermore, the high level of genome structure shared between zebrafish and humans (~70% of human genes have at least one obvious zebrafish ortholog, compared to 80% of human genes with mouse orthologs)^14,15^ has facilitated the use of zebrafish for understanding human genetic diseases. Recent advancements in next-generation sequencing (NGS) coupled with the demand for personalized medicine has further driven zebrafish uses in identifying causal relationships between the genotype and phenotype of various human diseases.

Additionally, zebrafish possess several advantages over rodent models in the study of vertebrate development and disease. These include hundreds of embryos in a single clutch and optical clarity of the developing embryo, which allows live imaging at the organism level^16,17^. In addition, the use of tissue-specific transgenic animals can be easily generated under the control of various selected gene promoters. Recent improvement of the Tol2-based transgenic system in zebrafish^18^ has allowed the control of gene expression in a spatiotemporal manner by coupling with regulatory elements such as GAL4/UAS or Cre/LoxP^19,20^. These advantages allow live imaging of cells and tracking of cellular dynamics in vivo to study the underlying molecular mechanisms of various developing organs.

The necessity of a model organism to recapitulate metabolic symptoms and associated disease development in humans has led to the exploitation of several animal species, among which rodents have been widely employed. For the past several decades, mice have been the leading experimental animal model in the field of biomedical research due to powerful genetic tools, amenable diagnostic parameters that are comparable to those in humans, and standardized protocols for developing, diagnosing, and treating metabolic syndromes. However, factors inherently different from those in humans, such as dietary requirements, lifestyle, and microbiomes, have called for alternative animal model systems to be utilized in parallel^21^. Zebrafish is a fascinating animal model for understanding the human pathogenesis of metabolic diseases and identifying potential therapeutic options^21^. However, all animal models have unique shortcomings, are the zebrafish model is no exception: first, zebrafish are poikilothermal animals living under water. Nonetheless, zebrafish possess metabolic characteristics similar to humans to complement data obtained from other model organisms, including rodents. This possibility has been clearly shown in recent studies^22–25^ in which drugs that had been approved for alleviating metabolic syndromes in humans were also effective in a zebrafish model.

This review addresses the use of zebrafish as an animal model for biomedical research, mainly in developmental disorders, mental disorders, and communication between the brain and organs. In addition to biomedical research, we also discuss the utility of zebrafish in metabolic control, focusing on cellular metabolic organelles.

## Biomedical research I: developmental disorders

During early animal development, an organizer can induce a complete body axis when transplanted to the ventral side of a host embryo. Studies have suggested that head inducers can inhibit Wnt signaling during the early development of anterior brain structures. In zebrafish, for example, it was demonstrated that head defects in the *headless* mutant were caused by a mutation in T-cell factor 3^5^. Loss of gene function in the *headless* mutant revealed that *headless* can repress Wnt target genes. These data provide the first genetic evidence that a component of the Wnt signaling pathway is essential in head/brain formation and patterning in vertebrate animals.

**Fig. 1:**
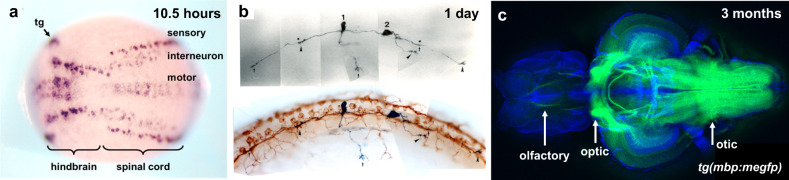
Development of the central nervous system in zebrafish. **a** Detection of early neuronal precursor cells by whole-mount in situ hybridization with a pan-neuronal marker, *huC*, at the neural plate stage (10.5 h after fertilization). Unpublished data. **b** Immunostaining of axonal growth in the spinal cord of one-day-old zebrafish. Double-staining with anti-gicerin antibody and anti-HNK-1 antibody^28^. **c** Confocal image of myelin structure in an isolated adult zebrafish brain visualized by *mbp* promoter-driven membrane-tagged GFP, *Tg(mbp:*mEGFP*)*. Arrows indicate the olfactory, optic, and otic nerves. Unpublished data.

Zebrafish have been a tractable animal model for identifying developing neurons and the in vivo architecture of the brain, from neurogenesis at the early neural plate stage to the adult brain (Fig. 1). The zebrafish HuC homolog, which is 89% identical to the human HuC protein, is one of the earliest discovered markers of neuronal precursor cells in zebrafish, which are apparent during neurogenesis as early as the neural plate stage (Fig. 1a)^26,27^. Zebrafish are useful for studying the functional role of novel genes in neuronal development through directed expression studies of the zebrafish nervous system (Fig. 1b)^28^. In addition, recently, tissue-clearing technology has allowed visualization of neural networks in the whole brain of adult zebrafish (Fig. 1c). These molecular tools and technologies are useful for investigating phenotypic changes in zebrafish disease models of human developmental disorders.

The Genome-wide Association Study (GWAS) investigates a genomewide set of variants in human genetic diseases to identify the causative gene variant associated with a particular disease. GWAS data can be used to identify single-nucleotide polymorphisms (SNPs) and other variants in the genome associated with genetic disease^29^. In contrast to the identification of SNPs and variants, phenotypic abnormalities and haploinsufficiency of the various genes are derived from microdeletions or chromosomal translocation of different genomes^30,31^. For instance, Potocki-Shaffer syndrome (PSS) is a disorder that affects the development of bones, nerve cells in the brain, and other tissues due to the interstitial deletion of band p11.2 in chromosome 11^32^. Developmental disorders in PSS were investigated using *phf21a*-knockdown zebrafish, producing developmental abnormalities in the head, face, and jaw, in addition to increased neuronal apoptosis^33^. Another example of a disease studied by zebrafish models is Miles–Carpenter syndrome (MCS), in which syndromic X-linked intellectual disability is characterized by severe intellectual deficit, microcephaly, exotropia, distal muscle wasting, and low digital arches. By whole-exome sequencing of MCS families, *ZC4H2* was identified as an MCS gene candidate. *ZC4H2*, a zinc-finger protein, is located in Xq11.2, and point mutations in *ZC4H2* were found in MCS patients. Homozygous *zc4h2*-knockout zebrafish larvae showed motor hyperactivity, abnormal swimming, and continuous jaw movement. Motor hyperactivity was caused by a reduction in V2 GABAergic interneurons, arising from misspecification of neural progenitors in the brain and spinal cord of the *zc4h2*-knockout zebrafish^34^. The knockout animals also exhibited contractures of the pectoral fins and abnormal eye positioning, suggestive of exotropia, indicating that zebrafish disease models can be used to study the underlying cellular and molecular mechanisms of human developmental disorders.

## Biomedical research II: mental disorders

Mental disorders, also called psychiatric disorders, are characterized by defective behavioral or mental patterns that cause significant distress to the subject. The International Classification of Diseases (ICD) published by the World Health Organization (WHO) is the international standard for classifying medical disease conditions. Over 450 different definitions of mental disorders are represented in the Diagnostic and Statistical Manual of Mental Disorders (DSM), the standard reference for psychiatry published by the American Psychiatric Association. Zebrafish are highly social animals that exhibit shoaling and schooling behaviors and are suitable for social behavioral tests in relation to mental disorders. Using mutagenesis screening, Kim and colleagues recently identified a novel chemokine-like gene family, *samdori* (*sam*), involved in mental disorders in zebrafish. Among the five *sam* family members, *sam2* is specifically expressed in the habenular nuclei of the brain and is associated with intellectual disability and autism spectrum disorder^35,36^. *Sam2*-knockout animals (both zebrafish and mouse) showed defects in emotional responses, such as fear and anxiety, that are involved in anxiety-related disorders and/or autism^35,36^.

Additionally, by whole-exome sequencing, *FAM50A* was identified as the causative gene for Armfield X-linked intellectual disability (XLID) syndrome. XLID refers to forms of mental disorders with intellectual disability that are explicitly associated with X-linked recessive inheritance. Approximately 100 genes have been found to be involved in XLID syndrome^37^. XLID accounts for ~16% of all cases of intellectual disability in males, who are more likely to be affected than females. The biological activity of human *FAM50A* missense variants was functionally validated by rescue experiments in a zebrafish *fam50a*-knockout model^38^. Using the zebrafish disease model, it was recently found that Armfield XLID syndrome is a spliceosomopathy associated with aberrant mRNA processing during development^38^.

## Biomedical research III: communication between the brain and other organs

Human puberty is a dynamic process that initiates the complex interactions of the hypothalamic-pituitary-gonadal axis (HPG axis), which refers to single endocrine glands as individual entities. The HPG axis plays a critical role in developing and regulating many of the body’s systems, particularly reproduction^39^. Gonadotropin-releasing hormone (GnRH), secreted by the hypothalamus in the brain, circulates through the anterior portion of the pituitary hypophyseal portal system and binds to receptors on the secretory cells of the adenohypophysis^40^. In response to GnRH stimulation, these cells produce luteinizing hormone and follicle-stimulating hormone, which circulate in the bloodstream^41^. Therefore, an adolescent develops into a mature adult with a body capable of sexual reproduction^42^. Kallmann syndrome (KS) is a genetic disorder known to prevent a person from starting or fully completing puberty. In a study showing that the *WDR11* gene mutation is involved in KS pathogenicity, the zebrafish *wdr11* gene was demonstrated to be expressed in the brain region, indicating a potential role for WDR11-EMX1 protein interaction^43^.

Additionally, acute inflammation is known to initiate regenerative response after traumatic injury in the adult zebrafish brain. The cysteinyl leukotriene receptor 1 (*cysltr1*)–leukotriene C4 (*LTC4*) pathway is required and sufficient for enhanced proliferation and neurogenesis^44^. LTC4, one of the ligands for CysLT1, binds to its receptor Cysltr1 expressed on radial glial cells in the zebrafish brain^44^. In a study by Kyritsis et al., *cysltr1* was increasingly expressed on radial glial cells after traumatic brain injury, suggesting cross talk between components of the inflammatory response and the central nervous system during traumatic brain injury^44^.

The nicotinamide adenine dinucleotide phosphate (NADPH) oxidase (NOX) family is involved in the production of reactive oxygen species in response to various extracellular signals. The NOX family member dual oxidase (DUOX) was identified as thyroid NADPH oxidase. In humans, DUOX2 mutations were identified among children diagnosed with congenital hypothyroidism. Recently, it was demonstrated that, in addition to goitrous thyroid glands and growth retardation, defects in anxiety response and social interaction were found in *duox*-knockout zebrafish^45^. These results suggest that *duox*-knockout zebrafish could serve as an effective animal model for studies in thyroid development and related neurological diseases, including intellectual disability and autism.

A large percentage of children with ASD are known to have gastrointestinal problems, such as constipation, diarrhea, and abdominal pain. Recent studies on the brain-gut axis have also shown that interactions with host-associated microbial communities, either directly by microbial metabolites or indirectly via immune, metabolic or endocrine systems, can act as sources of environmental cues. Molecular signals from the gut provide environmental cues for communication between the gut and the brain during episodes related to anxiety, depression, cognition or autism spectrum disorder (ASD)^46^. Moreover, modulation of intrinsic signaling pathways and extrinsic cues in resident intestinal bacteria enhances the stability of β-catenin in intestinal epithelial cells, promoting cell proliferation^47^.

## Biomedical research IV: metabolic disorders

### Zebrafish an animal models for metabolic research

A high-calorie diet, a sedentary lifestyle, and a family history of metabolic disorders increase the prevalence of risk factors such as low HDL levels, high triglyceride levels, high blood glucose, high blood pressure, and abdominal obesity^48^. Such metabolic disorders may arise from an imbalance between nutritional intake and energy expenditure, leading to the development of serious illnesses, including diabetes, stroke, and fatty liver disease^49^.

In addition to general similarities with human metabolism, zebrafish metabolism also exhibits unique characteristics. Zebrafish embryos consume yolk for the first five days of development, after which they are fed for further growth to prevent them from undergoing fasting. The feeding-to-fasting transition at 5–6 days post fertilization (dpf) has been utilized to develop mechanistic insights into metabolic homeostasis upon energy deprivation^50,51^. Another unique feature of zebrafish is the composition and development of adipose tissue. As a poikilothermal animal, zebrafish do not seem to require brown adipose tissue, on which mammals do depend. Adipose development occurs late in development, with the first adipocyte being detected 8 dpf^50^.

Interestingly, late adipogenesis may also provide an experimental setting by which the role of adipose tissue in the pathogenesis of metabolic disorders can be investigated. Modeling metabolism to recapitulate human disorders can be appropriately established during the larval period. Similarly, metabolic disorders can be modeled in adults to explore phenotype references in the presence of all major metabolic organs. Many metabolic similarities and discrepancies between humans and zebrafish and the modeling of different types of metabolic diseases have been reviewed elsewhere^52–54^.

### Zebrafish models for organelle biology research

Body metabolism is regulated by metabolic organelles, such as the endoplasmic reticulum (ER), mitochondria, peroxisomes, lipid droplets, and lysosomes. Whole-body metabolism is the sum of all metabolic activity of individual organs that originates from the metabolic function of individual cells. The function of subcellular organelles is critical for responding to environmental changes and regulating metabolic outputs to maintain metabolic homeostasis.

Zebrafish have served as an excellent model system to assess in vivo toxicity in response to treatment of a chemical of interest, and numerous studies have illustrated metabolic changes related to mitochondrial function upon chemical treatment^55–57^. After an initial study of mitochondrial activity and distribution in zebrafish oocytes reported in 1980, many reports regarding the mitochondrial genome and functional homologs of mitochondrial proteins in zebrafish were published in the late 1990s and early 2000s. More recently, zebrafish models have drawn extensive interest for use in testing a range of bioactive chemicals, including those that induce or disrupt development, improve disease conditions, or induce unfavorable side effects in daily human health or anticancer treatments^58–68^. In addition, the use of zebrafish as in vivo models for studying gene functions involved in metabolic activities has recently increased. Among the new molecular tools in developmental genetics, CRISPR/Cas9 is the most recent example of a reverse genetics technique, and mechanistic studies of the regulation of biogenesis, degradation and the quality maintenance of an organelle of interest have been conducted using zebrafish models^69^.

### CRISPR/Cas9 is the most advanced gene editing system

Recent findings and the development of CRISPR/Cas9, evolutionary gene-editing machinery that originated from the defense system of bacteria that earned its developers the Nobel Prize in Chemistry in 2020. Highly efficient gene targeting made it possible to edit a gene of interest in any genome. Accordingly, studies utilizing CRISPR/Cas9 in zebrafish have rapidly increased. In particular, studies to elucidate the role of mitochondria in neutrophil motility^70^, tRNA biogenesis and the physiology of cardiomyocytes^71,72^, neuronal regeneration^73^, neurodegeneration in Parkinson’s disease^74,75^ and cellular metabolism regulation of mitochondrial abundance^76,77^ have been reported. Furthermore, studies illustrating the role of the endoplasmic (sarcoplasmic) reticulum included *REEP5*-gene knockout, which was used to elucidate the previously unknown regulation of ER/SR membrane protein organization and stress response in cardiac myocytes^78^. In addition, the demonstration of MCTP (multiple C2 domain proteins with two transmembrane regions) gene function acting as a novel ER calcium sensor was also reported^76^.

Moreover, molecular pathogenesis studies based on the analysis of genes, such as *ATP13a*^79^, *NPC1*^80,81^, and *GBA1*^82^ to understand Niewmann-Pick disease type C1 (NPC1) and other lysosomal storage diseases resulting from defective intracellular trafficking or lysosome function have been reported. Efforts have also been made to elucidate molecules and regulatory mechanisms leading to autophagosome formation, autolysosome formation, and autophagy^83–86^. Recently, a possible knock-in strategy to edit mitochondrial DNA and genomic DNA has been reported^87^, facilitating research on organelle function in metabolic diseases.

### Transgenic approach to track organelle dynamics, abundance, and interaction

**Fig. 2:**
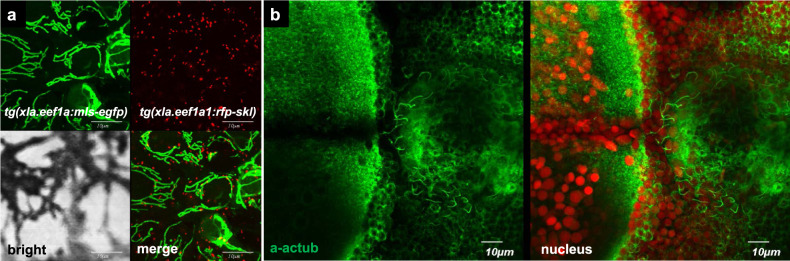
Subcellular organelles in the developing zebrafish embryos. **a** Using transgenic zebrafish lines 5 dpf, mitochondria, *Tg(Xla.Eef1a:*MLS-EGFP*)*, and peroxisomes, *Tg(Xla. Eef1a:*RFP-SKL*)*, in the skin of the developing larva are visualized. **b** Motile cilia (green) in the hindbrain 4th ventricle are visualized with anti-acetylated tubulin antibody, and nuclei are shown in red. Unpublished data.

Mitochondria have long been foci due to their roles in bioenergetics and apoptosis, leading to a plethora of transgenic zebrafish. Several transgenic zebrafish, such as Mnx1:MITO-Kaede^88^, hspa8:MITO-YC2^89^, and MLS-EGFP^90^, have been generated to mark mitochondria with fluorescent proteins GFP, YFP, Kaede, and yellow cameleon (YC), which are induced explicitly by a pan-expression promoter, an inducible heat shock promoter, a cell-type-specific promoter, or a combination of the GAL4-UAS system and are localized to the mitochondria using a mitochondria-targeting sequence^91^. One of the best examples of live mitochondrial imaging was illustrated in sensory axons of Rohon-Beard neurons, in which mitochondrial shape, dynamics, and transport were analyzed quantitatively^92^. A similar in vivo technique using zebrafish has since become popular to demonstrate the connection between mitochondrial behavior and neuronal health^93,94^. In addition to mitochondria, other organelles have been studied to reveal their roles during zebrafish development. For instance, a peroxisomal solute carrier, *slc25a17*, is involved in the maintenance of functional peroxisomes by showing substrate specificity towards coenzyme A^95^. To visualize peroxisomes in zebrafish embryos in vivo, the transgenic line *Tg(Xla.Eef1a:RFP-SKL)* was established and used under different metabolic conditions^96^. The use of double transgenic zebrafish allows simultaneous tracking of the dynamics of mitochondria and peroxisomes in vivo, as shown in Fig. 2a.

Another example is a transgenic line that marks the Golgi apparatus using the Golgi-Venus together with a cis-Golgi marker, GM-130^97^, to elucidate its role in dendrite specification of Purkinje cells. A trans-Golgi marker, GalT-GFP, was also established to reveal the dynamic localization of a connexin variant that influences cellular behavior^98^. A more systematic approach was applied to the study of secretory pathways, where a series of transgenic lines were generated based on different Rab proteins marking different types of endosomal vesicles^99^. A handful of transgenic lines were added to improve the identification of the cellular secretory pathways, and Lamp2-EGFP was used to mark lysosome-related vacuoles in the zebrafish notochord, GFP-CaaX (mem-GFP) was used to visualize the plasma membrane^100^, and NLS-mCherry or NLS-EGFP was used to the identify nucleus^101,102^. Another transgenic zebrafish used to mark the apoptotic cell membrane specifically, Annexin-Cy5, was also generated^103^. Moreover, transgenic zebrafish can be used to visualize transient and dynamic structures, with EGFP-LC3 used to monitor phagophore formation during autophagy^104^, Kif17-GFP^105^ used to analyze vesicles trafficking towards microtubule plus-ends, and EB1-GFP^106^ or EB2-GFP used to view microtubules growing in the plus-end. In combination with vital dyes, these transgenic zebrafish have been utilized extensively to advance our understanding of the dynamics of subcellular structures under physiological conditions and during pathological progression^107^.

### Bioimaging tools that enable in vivo analysis

**Fig. 3:**
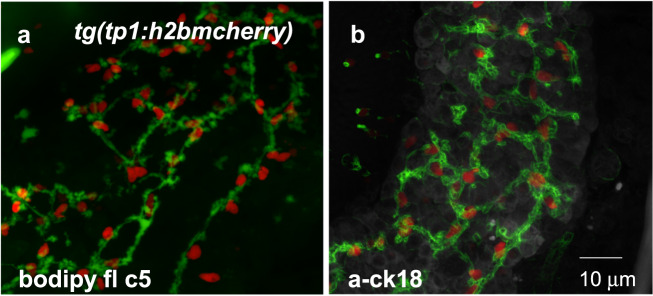
Bile duct formation in the developing zebrafish liver 6 dpf. **a**, **b** Using a transgenic zebrafish line, *Tg(Tp1:H2BmCherry)*, biliary epithelial cell nuclei are labeled red. The bile duct in the developing liver is visualized using the BODIPY FL-C5 dye (**a**) or the anti-cytokeratin 18 antibody (**b**). Unpublished data.

Advanced imaging tools that allow the examination of subcellular structures may facilitate the identification of previously unknown processes. These processes include communication between organelles upon membrane contact^108^, organelle biogenesis (peroxisome biogenesis^109^), organelle dynamics responding to an environmental cue^110^ and organelle trafficking along microtubules^111^. Notably, recent advances in microscopy have greatly enhanced the ability to observe cells in their native state and even monitor in vivo dynamics of organelles as well as ductal structure in the liver in zebrafish^110,112^. Motile cilia in the 4th ventricle of the hindbrain and bile duct of the developing liver can be visualized under confocal microscopy after specimens are immunostained with anti-acetylated tubulin (Fig. 2b) and with anti-cytokeratin 18 antibody (Fig. 3), respectively. High-speed, high-resolution, 3-dimensional in vivo imaging has enabled the dissection of dynamic intracellular processes and cellular behavior in response to different environments, which can enable the prediction of physiological conditions at the organism level. In this regard, a drug discovery platform based on organelle biology in zebrafish may play an essential role in the development of precision medicine and next-generation disease therapy.

## Conclusion

In summary, the zebrafish is a very useful vertebrate animal model in biomedical research and drug discovery. In particular, with the aid of CRISPR-based-knockout technology and big data from next-generation DNA sequencing, functional validation of GWAS candidates in zebrafish is greatly enhancing the ability and accuracy of identifying causative genes and molecular mechanisms underlying the pathogenesis of human genetic diseases. These efforts are fundamental to the establishment of a platform for the future of precision medicine, providing new molecular targets for diagnostic and therapeutic strategies, especially those involving rare diseases.
